# LgR5 expression and cancer stem cell hypothesis: clue to define the true origin of esophageal adenocarcinomas with and without Barrett's Esophagus?

**DOI:** 10.1186/1756-9966-30-23

**Published:** 2011-02-23

**Authors:** Burkhard HA von Rahden, Stefan Kircher, Maria Lazariotou, Christoph Reiber, Luisa Stuermer, Christoph Otto, Christoph T Germer, Martin Grimm

**Affiliations:** 1 Department of General-, Visceral-, Vascular and Pediatric Surgery, University Hospital Wuerzburg, Oberduerrbacher Strasse 6, 97080 Wuerzburg, Germany; 2 Institute of Pathology, University of Wuerzburg, Josef-Schneider Strasse 2, 97080 Wuerzburg, Germany; 3 Department of Cardiac and Thoracic Surgery, University of Wuerzburg Hospital, Oberduerrbacher Strasse 6, 97080 Wuerzburg, Germany

## Abstract

**Background:**

Investigation of the expression of an intestinal stem cell marker in esophageal adenocarcinomas (EAC) with and without Barrett's Esophagus (BE), with respect to a cancer stem cell (CSC) hypothesis.

**Materials and methods:**

Expression of a putative intestinal stem cell marker LgR5 was analyzed in esophageal cancer specimen (n = 70: 41 EAC with BE, 19 EAC without BE, and n = 10 esophageal squamous-cell carcinomas, ESCC) and in the adenocarcinoma cell line OE-33. Ki-67 and Cdx-2 were co-labelled with LgR5 in double staining experiments. Immunhistochemical expression results were confirmed by RT-PCR and correlated with tumor stage and five-year survival rates.

**Results:**

LgR5was found expressed in 35 of 41 (85%) EAC with BE and in 16 of 19 (81%) EAC without BE. By contrast, LgR5 was not found to be expressed in ESCC. Quantification of immunolabeling showed 15% LgR5+ cells in EAC with BE, 32% LgR5+ cells in adjacent BE and 13% in EAC without BE. Immunofluorescence double staining experiments with LgR5 and Ki-67 revealed a subpopulation (~5%) of proliferating LgR+/Ki-67+ cells. On mRNA-level, expression of LgR5 was higher in BE in comparison to EAC (p = 0.0159). High levels of LgR5 expression in BE associated EAC were associated with poorer survival in univariate analysis.

**Conclusion:**

The stem cell marker LgR5 is expressed in EAC, irrespective of association with BE, and appears to have negative impact on survival. The subset of proliferating LgR5+ cells (<5%) might resemble rapidly cycling CSCs, which needs to be substantiated in further investigations.

## Introduction

Esophageal adenocarcinoma (EAC) is an entity of increasing clinical importance, due to an unexplained incidence rise among white males in the Western world [1], and a dismal prognosis [2,3]. Chances for cure are still limited to early, surgically resectable tumor stages, prior to systemic dissemination of the disease. EACs develop almost exclusively in the distal third of the esophagus, under the chronically damaging effect of gastroesophageal reflux [2,3]. Barrett's esophagus (BE) - defined as columnar-lined epithelium in the distal esophagus, characterized by specialized intestinal mucosa (with goblet cells) - is regarded as a precancerous lesion, giving rise to these tumors.

Malignant progression within BE is regarded to follow a sequence of well-characterized histopathologic changes, from intestinal metaplasia, over low-grade and high-grade dysplasia/intraepithelial neoplasia towards invasive adenocarcinomas [2,3]. However, not all esophageal adenocarcinomas are associated with BE in surgical series [4,5], and only a minority of patients with BE progress to cancer, with an incidence of 0.5% per year [6]. These and other findings have raised doubt about the relevance of BE as precancerous lesion of EACs (e.g. [7]), stimulation the search for the cell population, from which EACs originate and which is currently unknown.

Two cancer models have been put forward to explain tumor heterogeneity and inherent differences of tumor-regenerating capacity [8]. The *clonal selection model *of carcinogenesis implies that a random solitary cell undergoes malignant transformation, accumulates multiple mutations and subsequently acquires a survival advantage, which leads to clonal selection [9,10]. In contrast, the *cancer stem cell (CSC) hypothesis *regards malignant transformation as a process, occurring in a subset of normal stem cells with pluripotent properties, which underlie deregulation of self-renewal pathways [11,12].

Evidence is accumulating that most, if not all, malignancies are driven by a cancer stem cell compartment [8]. The existence of cancer stem cells would explain why only a small minority of cancer cells is capable of extensive proliferation within the tumor. Furthermore, these cancer stem cells may be inherently resistant to our current therapeutic approaches. It is important to note that the two models are not mutually exclusive, as CSCs themselves may undergo clonal evolution, as already shown for leukaemia cells [13,14].

A stem cell hypothesis for BE has also been put forward by the group around Spechler [13]. It has been proposed that specialized intestinal metaplasia could arise from a change in the differentiation pattern of stem cells that might either reside in the esophagus or which might be recruited to the esophagus from the bone marrow [13]. A putative intestinal stem cell marker has been proposed to be potentially implicated in carcinogenesis of BE and EAC, but have so far not been thoroughly investigated. *Leucine-rich-repeat-containing G-protein-coupled receptor (LgR5) *has been shown to be associated with intestinal stem cell properties [15-18].

The aim of our study was to investigate expression of this putative intestinal stem cell marker in esophageal adenocarcinomas (EAC) with and without associated intestinal metaplasia (BE) as well as associated BE and squamous cell carcinomas. We aimed to give an indication for the carcinogenic process of EACs with respect to a cancer stem cell (CSC) hypothesis.

## Materials and methods

### Patients and Tumor Specimen

Surgical specimen from altogether 70 patients having undergone primary surgical resection for esophageal cancer between January 2001 and June 2004 with complete (R0) resection, were included in our study. Patients with preoperative antineoplastic therapies (chemoradiation/chemotherapy) were excluded.

The material was archival formalin-fixed, paraffin-embedded tissue from routine histopathologic work-up. Formalin-fixation and paraffin-embedding had been performed under standardized conditions. The material had been stored with permission of the local ethics committee, after informed consent obtained from the patients prior to surgical resection.

There were n = 41 esophageal adenocarcinomas (EAC) with associated Barrett's esophagus (BE), n = 19 EAC without BE and n = 10 esophageal squamous-cell carcinomas (ESCC) of the esophagus (which were included as negative controls). EAC without BE was defined based on clinical information (endoscopic evidence of Barrett's mucosa), work-up of all tumor blocks (specialized intestinal metaplasia) and Cdx-2 staining which is regarded to have a 70% sensitivity [19]. Of note, EAC were tumors in the distal esophagus (AEG type I tumors, according to the classification by Siewert and Stein, 1998, Br J Surg [20]), and explicitly not localized at the level of the anatomic gastric cardia (AEG type II tumors). The AEG type II adenocarcinoma is a tumor entity on its own and must be discussed differently.

Follow-up data were obtained from our local tumor registry of Lower Frankonia/Germany and was complete (100%) for all patients. In this tumor registry, data are stored also with permission obtained from the patients and due to the regulation of the local ethics committee. Mean follow-up was 29 months ± 17.6 standard deviation. Tumor and patient characteristics are summarized in Table 1 and 2.

**Table 1:** Clinicopathological characteristics of the EAC study population with BE

Characteristics	Patients (n = 41)	LgR5 Barrett's esophagus*		p-value	LgR5 Barrett's EAC		p-value
		low	high		low	high	
Age (y)				.100			.051
<66	21 (51%)	10 (48%)	11 (52%)		4 (19%)	17 (81%)	
≥66	20 (49%)	4 (20%)	16 (80%)		10 (50%)	10 (50%)	
Gender				.074			.673
Male	34 (83%)	14 (41%)	20 (59%)		11 (32%)	23 (68%)	
Female	7 (17%)	0 (0%)	7 (100%)		3 (43%)	4 (57%)	
Histological Grading				.305^a^			.083^a^
G1	11 (27%)	6 (55%)	5 (45%)		6 (55%)	5 (45%)	
G2	16 (39%)	5 (31%)	11 (69%)		6 (37%)	10 (63%)	
G3/4	14(34%)	3 (21%)	11 (79%)		2 (14%)	12 (86%)	
Depth of invasion				.481^b^			.155^b^
pT1	10 (24%)	4 (40%)	6 (60%)		6 (60%)	4 (40%)	
pT2	18 (44%)	7 (39%)	11 (61%)		6 (33%)	12 (67%)	
pT3	6 (15%)	1 (17%)	5 (83%)		1 (17%)	5 (83%)	
pT4	7 (17%)	2 (28%)	5 (72%)		1 (14%)	6 (86%)	
Lymph node metastases				.001			.0154
pN0	15 (37%)	10 (67%)	5 (33%)		9 (60%)	6 (40%)	
pN1-3	26 (63%)	4 (15%)	22 (85%)		5 (19%)	21 (81%)	
UICC stage				.481^c^			.155^c^
UICC I	9 (22%)	4 (44%)	5 (56%)		2 (22%)	4 (78%)	
UICC II	19 (46%)	7 (37%)	12 (63%)		7 (37%)	12 (63%)	
UICC III	13 (31%)	3 (23%)	10 (77%)		5 (15%)	11 (85%)	
UICC IV	0 (0%)	0 (0%)	0 (0%)		0 (0%)	0 (0%)	
Median OS (m)	42 m	32 (n = 14)	24 (n = 27)		33 (n = 14)	28 (n = 27)	

Abbrevations: EAC, esophageal adenocarcinomas; BE, Barrett metaplasia; y, years; G, grading; UICC, International Union against Cancer; R, residual tumor; OS, overall survival; m, months. *Clinico-pathological features of BE are related to adjacent EAC.

^a^G1/2 vs. GT3/4; ^b^pT1/2 vs. pT3/4; ^c^UICC I/II vs. UICC III/IV

**Table 2:** Clinicopathological characteristics of the EAC study population (with and without BE)

Characteristics	Patients (n = 60)	LgR5 EAC		p-value
		low	high	
Age (y)				.069
<66	30 (50%)	10 (33%)	20 (67%)	
≥66	30 (50%)	18 (60%)	12 (40%)	
Gender				1.00
Male	52 (87%)	24 (46%)	28 (34%)	
Female	8 (13%)	4 (50%)	4 (50%)	
Histological classification				.577^a^
G1	17 (28%)	11 (65%)	6 (35%)	
G2	22 (37%)	11 (50%)	11 (50%)	
G3/4	21 (33%)	6 (29%)	15 (71%)	
Depth of invasion				.259^b^
pT1	16 (27%)	11 (69%)	5 (31%)	
pT2	26 (43%)	11 (42%)	15 (58%)	
pT3	10 (17%)	4 (40%)	6 (60%)	
pT4	8 (13%)	2 (25%)	6 (75%)	
Lymph nodes metastasis				.007
pN0	23 (38%)	16 (70%)	7 (30%)	
pN1-3	37 (62%)	12 (32%)	25 (68%)	
UICC stage				.573^c^
UICC I	14 (23%)	10 (71%)	4 (29%)	
UICC II	28 (47%)	11 (39%)	17 (61%)	
UICC III	18 (30%)	7 (39%)	11 (61%)	
UICC IV	0 (0%)	0 (0%)	0 (0%)	
Median OS (m)	43 m	32 (n = 28)	24 (n = 32)	

Abbrevations: EAC, esophageal adenocarcinomas; BE, Barrett metaplasia; y, years; G, grading; UICC, International Union against Cancer; R, residual tumor; OS, overall survival; m, months.

^a^G1/2 vs. GT3/4; ^b^pT1/2 vs. pT3/4; ^c^UICC I/II vs. UICC III/IV

### Histopathologic Analysis, Tumor Staging and Definition of Barrett's mucosa

Tumor blocks of paraffin-embedded tissue were selected by two experienced gastrointestinal pathologists (Stefan Kircher, Stefan Gattenlöhner), evaluating the routine H.E. stained sections. Sections from all available tumors underwent intensive histopathologic assessment, blinded to the prior histopathology report. H.E. stained sections were analyzed with respect to tumor infiltrated areas (EAC/ESCC), stromal areas and infiltrating immune cells. Tumor staging was performed according to the 6^th ^edition of the TNM staging system by the UICC/AJCC of 2002 [21]. Grading was performed according to WHO criteria [22]. Tumor characteristics (UICC stage, pT-categories, pN-categories, cM-categories, number of removed lymph nodes, number of tumor infiltrated lymph nodes, residual tumor status, localization) and patient characteristics were collected in a database (EXCEL, Microsoft).

Barrett's muscosa was defined as specialized intestinal metaplasia, with goblet cells [2,3]. In addition, immunohistochemistry with Caudal type homeobox transcription factor 2 (Cdx-2), which is suggested as early marker for intestinal metaplasia [23] with a known sensitivity of 70% [19], was used to identify tiny foci of intestinal metaplasia. Furthermore, different degrees of high-grade and low-grade intraepithelial neoplasia within Barrett's mucosa were assessed. EAC were classified as "EAC with BE", when at least tiny foci of intestinal metaplasia were found due to Cdx-2 staining. EAC were classified as "EAC without BE", when the pathologists could not find intestinal metaplasia on any of the tumor blocks.

### Immunohistochemical and immunofluorescence staining

Staining for LgR5, Cdx-2, and Ki-67 was performed on serial sections of 2 μm thickness. Tissue sections were cut from formalin-fixed paraffin-embedded (FFPE) blocks on a microtome and mounted on adhesive microscope slides (Hartenstein, Wuerzburg, Germany).

For immunohistochemistry, unconjugated polyclonal LgR5 (rabbit), and isotype control antibodies (mouse, rabbit) were purchased from Abcam (Cambrige, UK). The unconjugated mouse monoclonal Cdx-2 antibody was obtained from Biogenex (San Ramon, USA) and the unconjugated mouse monoclonal Ki-67 antibody was purchased from Acris (Hiddenhausen, Germany). The secondary antibody used for immunofluorescence double staining of Ki-67 was a fluoresceinisothiocyanat (FITC)-conjugated AffiniPure donkey-anti-mouse IgG, used at 1:200 dilution (Jackson ImmunoResearch Laboratories Inc., Suffolk, England). The secondary antibody for LgR5 was a Cy3-conjugated AffiniPure donkey-anti-rabbit IgG (Jackson ImmunoResearch), used at 1:200 dilution.

Normal colon tissue was used as positive control for LgR5 expression [24,25]. The colon tissue had undergone the same processing, like the esophageal cancer specimen (normal formalin-fixed, paraffin-embedded tissue from colon resections for benign conditions - normal colon mucosa adjacent to polyps or diverticular disease).

### Cell Culture

We analyzed LgR5 expression in cells (1 × 10^4^) from the esophageal adenocarcinoma cell line OE-33 (Sigma-Aldrich, Steinheim, Germany) in cytospins as additional positive control for LgR5 expression. This cell line is the only commercially available adenocarcinoma cell line of the lower esophagus (Barrett's metaplasia) and was established from a 73-year-old female patient. The tumor was identified as pathological stage IIA (UICC) and showed poor differentiation. Using RT-PCR we tested negative for mycoplasma contamination of this cell line that was provided to our laboratory in December 2009 by Sigma. The cell line was cultured in RPMI-1640 medium, supplemented with 10% Fetal Bovine Serum, 100 units/ml of penicillin and 100 μg/ml of streptomycin. Cytospins of the OE-33 cell line were fixed in acetone and dried for 10 minutes. Rehydration, blocking, and the staining procedure steps were the same as described for immunohistochemistry of FFPE sections. Additionally, RT-PCR was performed for LgR5 gene expression of OE-33 cells.

### Double Staining Experiments (IF and IHC)

The sequential immunofluorescence (IF) double staining (co-expression) was analyzed for LgR5 with Ki-67 expression. Sequential immunohistochemical (IHC) double staining was performed for Cdx-2 and LgR5.

### Processing of tissue and staining procedure

Serial tissue sections (2 μm thickness) were cut from formalin-fixed paraffin-embedded (FFPE) blocks on a microtome and mounted from warm water onto adhesive microscope slides (Hartenstein, Wuerzburg, Germany). Sections were deparaffinized in xylene and ethanol and rehydrated in water. Heat induced epitope retrieval (HIER) was performed with citrate buffer pH 6.0 (Dako, Hamburg, Germany). For immunofluorescence, slides were incubated in normal serum (2%) and bovine serum albumin (BSA 0.5%) at room temperature for 20 minutes to block non-specific binding. Subsequently, slides were incubated with the primary antibody or control antibody overnight at 4°C in a humidified chamber and with secondary FITC-conjugated antibody for 30 minutes at room temperature. Slides were subsequently incubated with the second primary antibody diluted in TBS plus 0.5% BSA overnight at 4°C in a humidified chamber followed by incubation with secondary Cy3-conjugated antibody for 30 minutes at room temperature in a humidified chamber. Slides were counterstained with DAPI (4',6-Diamidino-2-phenylindoldihydrochlorid) (Sigma-Aldrich) and covered with Polyvinyl-alcohol mounting medium (DABCO) (Sigma-Aldrich) and analyzed using a Zeiss camera (Jena, Germany). The photographed images - using the Metamorph software package (Visitron Systems, Puchheim, Germany) - were imported into the Microsoft Office Picture Manager.

For immunohistochemistry, the pretreatment procedure (fixation, deparaffinization, rehydration, HIER, and blocking) of the slides was the same as described for immunofluorescence. Endogenous peroxidase activity was quenched with 3% hydrogen peroxide. Endogenous biotin activity was blocked using the avidin/biotin blocking kit (Vector Laboratories, Burlingame, CA, USA). Slides were then incubated with the primary antibody alone (LgR5, Cdx-2, and Ki-67) or with pre-incubated (30 minutes) LgR5 blocking peptide (Abgent, San Diego, CA, USA) and LgR5 antibody. After incubation with the primary antibody the DAKO LSAB2 System, peroxidase, was used. Slides were subsequently incubated for 5 minutes in DAB (3,3'-diaminobenzidine) (Biogenex) counterstained with hemalaun and mounted with Glycergel (Dako). For immunohistochemical double staining, we first used an alkaline phosphatase (AP)-conjugated AffiniPure Donkey anti-mouse Ab followed by 20 minutes of incubation with Fast Red (Dako). After incubation with the second primary antibody, we used a horseradish peroxidase (HRP)-conjugated AffiniPure Donkey anti-rabbit IgG (Jackson ImmunoResearch) followed by 5 minutes of incubation with DAB (Biogenex). Cytospins were fixed in acetone and dried for 10 minutes. Rehydration, blocking, and the staining procedure was the same as described for immunohistochemistry of FFPE sections.

### Quantification of Immunohistochemistry and Immunofluorescence

LgR5 and Ki-67 IHC was quantified in EAC with BE, in the associated Barrett's mucosa, as well as EAC without BE. Quantification of immunoenzymatic staining of intestinal metaplasia or tumor cells was performed analyzing six defined representative individual high power fields (× 400) for each staining sample. Scoring was done by means of cell counting. The results were expressed as percentages (number of positive cells within 100 counted tumor cells, %). Sections were evaluated by two independent blinded investigators separately and, in case of discrepancies, both evaluated the slides simultaneously and made an agreement.

For each tumor section, quantification of immunofluorescence double staining was performed by counting Ki-67+ cells in six high power fields (400 × magnification) in parallel with LgR5+. The proportion of Ki-67 positivity in counted LgR5+ cells was expressed in percentages.

### Real-time quantitative reverse transcription-PCR analysis

To analyze gene expression of LgR5 by RT-PCR, we extracted total cellular RNA and performed cDNA synthesis using the Absolutely RNA FFPE Kit and AffinityScript QPCR cDNA Synthesis Kit from Stratagene (Waldbronn, Germany). Areas of interest (only epithelial regions) for each tissue section were manually microdissected using a scalpel blade. For both groups (BE and EAC without BE) equal amounts of tissue areas were assessed (2 × 1.5 cm^2 ^surface area per section, thickness of 10 μm). RNA extraction and cDNA synthesis were performed according to the manufacturer's instructions. For OE-33 cell line, after homogenization Diethyl pyrocarbonate (DEPC)-75% ethanol was added to the lysate to provide ideal binding conditions. Primers were designed using the Primer Express software for primer design to amplify short segments of 50-150 base pairs of target cDNA. The LgR5 forward primer sequence was: 5'-TGCTGGCTGGTGTGGATGCG-3'; the LgR5 reverse primer sequence was: 5'-GCCAGCAGGGCACAGAGCAA-3'. Matched human esophageal cDNA was purchased by BioChain (Hayward, CA, USA) as control. The housekeeping gene Glyceraldehyde-3-phosphate dehydrogenase (GAPDH) was used for relative quantification and cDNA quality control. The GAPDH forward primer sequence was: 5'-ATCCCATCACCATCTTCCAGG-3'; the GAPDH reverse primer sequence was: 5'-CGCCCCACTTGATTTTGG-3'. All PCR reactions were carried out with a DNA Engine Opticon 2 System (MJ Research, Biozym, Oldendorf, Germany). Total RNA was reversely transcribed into cDNA according to the manufacturer's manual. Each PCR reaction was performed in 25 μl volume containing 12.5 μl the Sensi Mix (Peqlab, Erlangen, Germany), 0.5 μl SYBR Green, 10 pmol/μl forward primer, 10 pmol/μl reverse primer, 1 μl template DNA (150 ng) and 9 μl peqgold RNAse free water. Initial denaturation at 95°C for 10 minutes was followed by 38 cycles of a denaturation step at 95°C for 15 seconds, an annealing step at 60.9 °C for 30 seconds, and an extension step at 72°C for 40 seconds. To confirm amplification specificity, the PCR products from each primer pair were subjected to a melting curve analysis. Negative controls without template were produced for each run.

Quantification data were analyzed using the LightCycler analysis software. Reproducibility was confirmed by independent PCR repeated twice. The average threshold cycle (Ct) value was calculated as the cycle number at which the fluorescence of the reporter reaches a fixed threshold. The difference (ΔCt) between the average Ct values of the samples in the target wells and those of the housekeeping gene (GAPDH) was assessed, followed by calculation of the difference between the average ΔCt values of the tumor samples for each target and the ΔCt value of the normal tissues for that target (ΔΔCt). The relative quantification value, fold difference, is expressed as 2^-ΔΔCt^.

### Statistical analysis

Statistical analysis was performed with MedCalc Software, Version 11.3.2 (Mariakerke, Belgium). All values were expressed as Median ± Interquartile Range (IQR) because a normal distribution of gene and protein expression could not be confirmed by the D'Agostino-Pearson test. Therefore, the Median value was chosen to divide patients in two different groups. Survival time was determined as the time from tumor resection to tumor conditional death and as the time from tumor resection to time of obvious recurrence. The overall survival (OS) time in association with LgR5 expression was estimated using the Kaplan-Meier method [26]. To analyze differences in the overall/tumor related survival among patients after successful (R0) curative surgical resection for EAC patients were divided into two subgroups (dichotomous variables). Median cut-off value for either high or low expressors was set at 33% for LgR5 expression in BE (n = 41), 15% for LgR5 expression in adjacent EAC (n = 41), and 15% for LgR5 expression in all EAC (n = 60); univariate analysis of significance for LgR5 expression differences in survival curves was evaluated with the log rank test. Multivariate with the Cox Proportional Hazards Model [27] was performed including all parameters that were found to be significant on univariate analysis. Fisher's exact test was used to investigate the relation between two categorical variables. Data were analyzed using the non-parametric Mann-Whitney U test or Kruskal-Wallis test when more than 2 groups were compared. P values of less than 0.05 were regarded statistically significant.

## Results

### LgR5 Immunohistochemistry

Immunohistochemistry against the putative intestinal stem cell marker LgR5 showed positive stainging in 85% (35 of 41) of the specimen of patients with EAC with BE, and 84% (16 of 19) in EAC without BE (p = n.s). No LgR5 expression was found in specimen with esophageal SCC.

No expression of the putative stem cell marker (LgR5) was detected in normal esophageal squamous cell epithelium, adjacent to the tumor.

**Figure 1:**
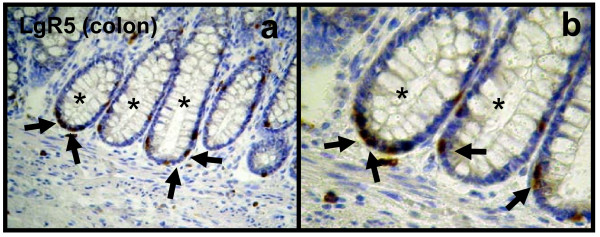
**Immunohistochemical staining of LgR5 (membranous staining pattern, brown) in normal colon tissue**. Normal colon mucosa (asterisk) showed the typical staining pattern as they stained the well-described putative colon mucosa stem cells, located at the basis of the crypts, or the transit amplifying zone, which are regarded to resemble the stem cell niche (arrows). Original magnification, × 200 (**a**) and × 400 (**b**).

Normal colon mucosa (used as positive control) showed the typical staining pattern of LgR5 (Figure 1a and 1b), as they stained the well-described putative colon mucosa stem cells, located at the basis of the crypts, or the transit amplifying zone, which are regarded to resemble the stem cell niche [24,25].

### Quantification of LgR5 Immunohistochemistry

**Figure 2:**
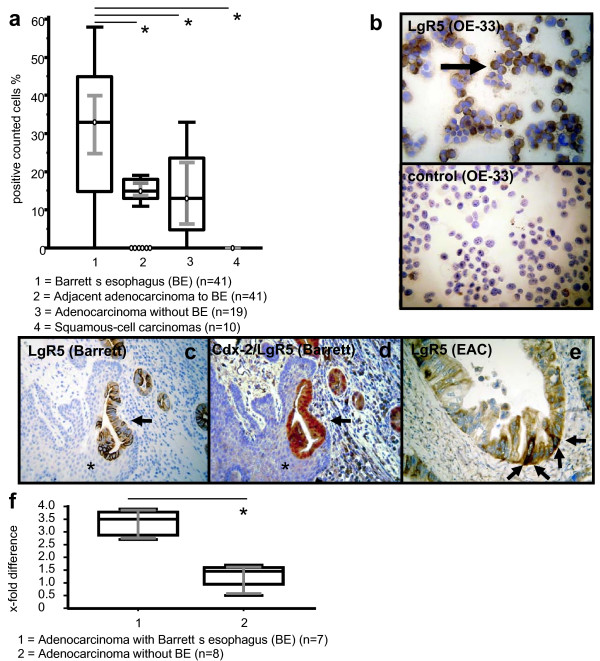
**Immunohistochemical analysis, staining and gene expression of LgR5**. In comparison to BE (1) a significantly (p < 0.05) decreased expression of LgR5 was observed in associated EACs (2) and EACs without BE (3). ESCC showed no LgR5 expression (4). Analysis refers to percentages of positivity of all counted cells. Grey lines show 95% confidence intervals. Statistically significant values from BE to EACs and ESCC are indicated with asterisks (**a**). LgR5 staining in cytospins from the OE-33 adenocarcinoma cancer cell line served as additional positive control (left, top) and showed 25% positive cells; Preincubation with LgR5 blocking peptide completely abolished LgR5 immunoreactivity (right, bottom) (**b**). Increased expression of LgR5 (**c**) was observed in early BE (arrows). Adjacent normal tissue stained negative for LgR5 (asterisk). Single staining of LgR5 in BE was confirmed by immunohistochemical double staining (**d**), showing Cdx-2 (nuclear staining pattern, Fast red) and LgR5 (membranous staining pattern, brown). Significantly decreased LgR5 expression was observed in adenocarcinomas compared to BE. Staining was observed in putative stem cell niches at the bottom of EACs (arrows) (**e**). Original magnification × 200. Gene expression of LgR5 in human BE and EAC (x-fold difference mRNA). LgR5 gene expression in BE-associated EAC (1) was significantly (p = 0.0159) higher in comparison to EAC without BE (2). Grey lines show 95% confidence intervals. Statistically significant value is indicated with an asterisk. Normal tissue is considered as one-fold (**f**).

Furthermore, we analyzed positivity of all counted cells according to the precursor lesion and tumor entity. LgR5 expression was significantly upregulated in BE (n = 41, Median 33%, IQR 14.75% - 45.0%; 95% CI 24.761 - 39.954%; p < 0.05; Figure 2a) but was decreased in adjacent EAC (n = 41, Median 15%, IQR 13.0% - 18.0%; 95% CI 13.761 - 17.0%; p < 0.05; Figure 2a) and EAC without BE (n = 19, Median 13%, IQR 4.75% - 23.0%; 95% CI 6.346 - 22.436%; p < 0.05; Figure 2a; p < 0.05 for LgR5 expression of BE with adjacent EAC and EAC with and without BE). No differences of LgR5 expression were found between different degrees in high-grade and low-grade intraepithelial neoplasia within Barrett's mucosa and did not significantly differ from EAC. Median LgR5 expression of all EACs (n = 60) was 15%, IQR 11.0% - 18.0%; 95% CI 13.0 - 16.061%. For adenocarcinomas without BE, the results of LgR5 expression were comparable with the lower expression levels of adenocarcinomas from BE (Figure 2a, Table 1 and 2). Stainings from the OE-33 adenocarcinoma cancer cell line in cytospins served as additional positive controls for LgR5 expression and showed 25% positive cells (Figure 2b). Preincubation with LgR5 blocking peptide completely abolished LgR5 immunoreactivity (Figure 1b).

Figure 2c and 2d demonstrate representative examples of LgR5 and Cdx-2 staining in early BE. We confirmed areas analyzed for LgR5 expression of BE by means of immunohistochemical co-labelling with Cdx-2 (Figure 2d). Staining was observed in putative stem cell niches at the bottom of BE and EACs (Figure 2e).

### LgR5 Gene Expression Analysis on mRNA Level

To confirm the results of the immunohistochemical staining, gene expression of LgR5 in human EAC was assessed on mRNA level by means of semiquantitative RT-PCR. EAC associated BE (Median 3.5-fold difference compared to normal tissue; IQR 3.025 - 3.725-fold difference; n = 7) exhibited LgR5 gene expression which was significantly (p = 0.0159) higher in comparison to EAC without BE (Median 1.4-fold difference compared to normal tissue; IQR 0.900 - 1.650-fold difference; n = 8; Figure 2f). These results confirmed increased LgR5 expression in BE adjacent to EAC and significantly decreased expression of LgR5 in EAC without BE as observed by immunohistochemistry. LgR5 RT-PCR results of the OE-33 adenocarinoma cell line showed 4.8-fold difference compared to normal tissue.

### LgR5 Expression in Relation to Proliferative Activity (Ki-67+)

**Figure 3:**
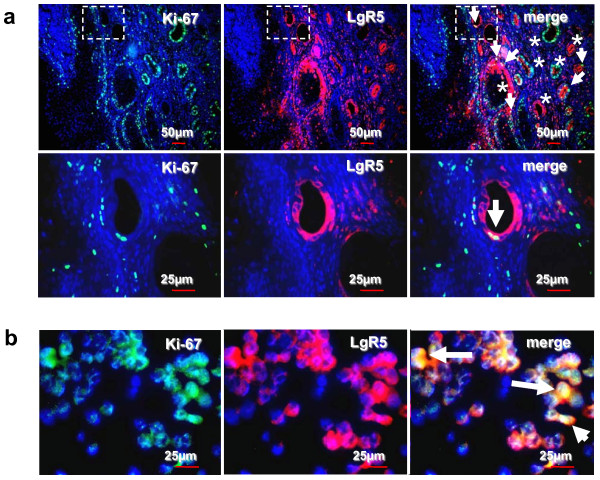
**Co-expression of LgR5 with Ki-67 in BE and OE-33 cells by immunofluorescence double staining**. Images demonstrate a representative example of LgR5 co-expression with Ki-67 in early BE showing positivity for a small subset of LgR5+ cells with Ki-67+ (arrows). In contrast, a dominant population of proliferating (Ki-67+) Barrett cells were LgR5-, which may drive multi-step carcinogenesis (asterisks). Vice versa, most of LgR5+ Barrett cells were Ki-67- (asterisks). Proliferating LgR5+ OE-33 cells (arrows) are shown below (b). FITC green Fluoresceinisothiocyanat, Cy3 red, and DAPI 4',6-Diamidino-2- phenylindoldihydrochlorid blue. Top (**a**), Calibration bar represents 50 μm. Bottom, Calibration bar represents 25 μm (**a **and **b**). Case demonstrates area of magnification.

For further investigation of the adoptive role of LgR5 in BE and its relation to potentially cancer-initiating cells in early BE, we analyzed proliferation status of LgR5 expressing early Barrett cells. A small subset of LgR5+ cells were Ki 67+ (proportion of Ki-67 positivity in counted LgR5+ cells was <5%). As shown in Figure 3a and 3b, Ki-67 was co-expressed with only a small subset of LgR5+ cells in areas which were associated with early BE (Cdx-2 positivity was observed in serial sections) (Figure 3a, representative example of n = 41 BE and associated adenocarcinomas) and OE-33 cells (Figure 3b). Vice versa, most of LgR5+ Barrett cells did not proliferate, as they did not exhibit nuclear staining with the proliferation marker (Ki-67-). In contrast, we analyzed a dominant population of proliferating Ki-67+/LgR5- cells (Figure 3a). Although down-regulated in EAC with BE, as well as EAC without BE, we confirmed a minority of proliferating cells in Cdx-2 negative (Cdx-2-) areas (data not shown).

### Prognostic Effect of LgR5

**Figure 4:**
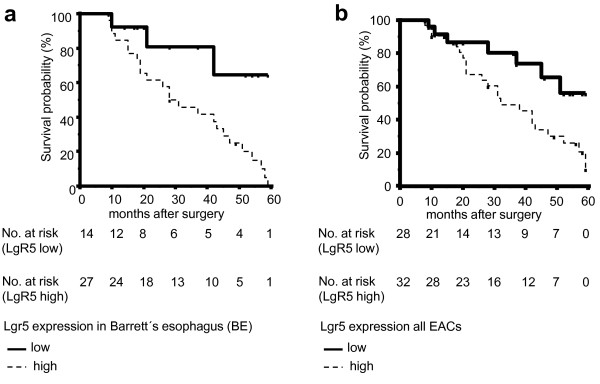
**Kaplan-Meier survival curves**. Overall survival curves calculated by Kaplan-Meier method in Barrett-associated EACs **(Figure 4a) **and the whole population of all EACs **(Figure 4b)**, respectively. Survival of patients with EAC was better when BE showed low LgR5 expression compared to high LgR5 expression. This was shown for BE in association with EAC (p = 0.0278) (**a**) and the whole population of EACs (**b**), respectively (p = 0.0325). The times of the censored data are indicated by short vertical lines.

To analyze survival differences of patients after successful (R0) curative surgical resection for EAC with and without BE, patients were divided into two subgroups as described above (dichotomous variables). Lymph node metastasis (pN+, p < 0.0001, Hazard Ratio (HR) = 12.1940, 95% CI = 5.9509 - 24.9867), pT-category (pT3/4, p < 0.0001, HR = 3.8447, 95% CI = 1.5309 - 9.6553) and grading (G3/4, p < 0.0001, HR = 4.0652, 95% CI = 1.7123 - 9.6514) were shown to be unfavorable factors in univariate analysis in the whole population of all EACs (n = 60). Survival in subgroup with high LgR5 expression in BE (n = 41, p = 0.0278, HR = 3.5145, 95% CI = 1.5050 - 8.2073, Figure 4a), adjacent EACs (n = 41, p = 0.039, HR = 2.8408, 95% CI = 1.2496 - 6.4582) and all EACs (n = 60, p = 0.0325, HR = 2.4175, 95% CI = 1.1719 - 4.9872, Figure 4b) was significantly poorer in comparison to the subgroup of patients with low expression of LgR5 (Table 1 and 2). Data suggest that LgR5 expression in BE and adjacent EACs is associated with clinical pathological features which may predict worse clinical outcome of related (adjacent) adenocarcinomas. Multivariate analysis using the Cox Proportional Hazards Model demonstrate lymph node metastasis and grading but not LgR5 expression as independent prognostic factors in all (n = 60) EACs (LN positive: Exp (b) 9.1861; 95% CI of Exp (b) 2.0665 - 40.8346; p = 0.003746. Grading G3/4: Exp (b) 2.2593; 95% CI of Exp (b) 1.0171 - 5.0186; p = 0.4643).

## Discussion

Similar to other solid tumor entities [8], a stem cell hypothesis has been proposed for Barrett's esophagus (BE) and its association with EAC [13]. However, this hypothesis has not undergone thorough investigation so far. An intestinal stem cell marker, LgR5 has been proposed [13], but have also not been thoroughly addressed in histogenetic studies.

Our results of LgR5 expression in EAC with and without BE, as well as the adjacent Barrett mucosa suggest that LgR5 might be a promising marker to further address the stem cell hypothesis. In esophageal SCC - as expected no LgR5 expression was found, which is due to the fact that ESCC is not derived from an intestinal (glandular) type epithelium.

Several studies have already focused on the effects of different LgR5 expression in the context of tumor development and progression. LgR5 has been demonstrated to be involved in the pathogenesis of different human cancer entities, including hepatocellular carcinoma [28], basal cell carcinoma [29], endometrial cancer [30], colon cancer and ovarian cancer [31]. Interestingly, LgR5 was identified to be expressed on crypt stem cells (precursor cells) as well as lesions which had progressed to cancer [15,32]. One previous study has demonstrated expression of LgR5+ in BE and EAC [33].

Our results of significant upregulation of LgR5 in BE and downregulation in associated EAC are in concordance to results in other solid tumor entities. In the endometrium, high expression of LgR5 is observed during the initial stages of tumorigenesis, but down-regulation of LgR5 is described for fully developed tumors [30]. This is well in line with our findings in EAC. Our results might be explained with the clonal selection model of carcinogenesis, which proposes that there is a subsequent clonal selection of putative stem cells [8]. The expression profile of LgR5 in EAC without BE was comparable with the result of EAC with BE.

According to a longstanding cancer model, known as the '*clonal evolution model'*, tumors arise from normal cells that mutate and generate abnormal offspring that do also mutate, forming a mass of genetically varied cancer cells. However, there has been a new wave of interest in an alternative explanation - that tumors are initiated and driven by a single, abnormal type of cancer stem cell, resulting in a population of genetically identical tumor cells. This is the '*cancer stem cell hypothesis' (CSC) *which is currently intensively discussed in the oncologic literature [8].

Our double-staining experiments, with the putative stem cell marker LgR5 and the proliferation marker Ki-67 demonstrated three different cell populations. First, a substantial fraction of cells was found to express the putative stem cell marker LgR5, which were not cycling (LgR5+/Ki-67-). These might be regarded as quiescent stem cells, or postmitotic dedifferentiated cells. Secondly, there was a major cellular compartment in BE as well as EAC, which showed no expression of the putative stem cell marker LgR5, but which were actively cycling (LgR5-/Ki-67+). This result might be interpreted in line with the clonal selection theory. If LgR5 marks stem cells, there are many of LgR5 negative non-stem cells, which are nevertheless cycling. Therefore a combination of clonal selection and cancer stem cell model, as previously suggested by others [8,34] might be applied. Moreover, we found a small subpopulation of cells within BE as well as esophageal AC, which expressed the putative stem cell marker LgR5, and which were actively cycling (LgR5+/Ki-67+). This population accounted for approximately 5% of BE. According to our hypothesis, that the intestinal stem cell marker LgR5 might also be suited to identify cancer stem cells, these might be the actively cycling Barrett (cancer) stem cells.

Our findings are in line with current cancer models [8] suggesting an integration of the CSC hypothesis and the clonal selection model [34]. CSCs may undergo clonal selection, thereby forming a second generation of CSCs, which may itself give rise to a tumor [8].

We assume that at least a portion of the proliferating population consists of LgR5+ Barrett cells and these results are compatible with the view that a minority population of Barrett cells is able to proliferate and contribute to the numbers of a larger Barrett cell population with a modified capacity for proliferation. Such a situation would be analogous to that found in normal hemopoietic differentiation, where a minority population of stem cells proliferates and gives rise to a large population of progeny, most of which have lost stem cell properties.

Finally, adenocarcinoma in BE may contain a cellular subcomponent that retains key stem cell properties [13,33,35,36]. Chronic activation of LgR5 expressed by BE in these putative pluripotent cancer-initiating cells may sustain inflammation responses, mediate resistance to apoptosis and promote further progression of the metaplasia - intraepithelial neoplasia - carcinoma sequence. Therefore targeting of LgR5 signalling might be a potential mechanism to abrogate this inflammation-mediated effect in tumor progression. This may be the reason for the higher expression of LgR5 in precancerous cells of BE, in comparison to cells of invasive AC. LgR5 signalling may therefore play a biological role in potentially cancer-initiating BE cells.

Although Barrett's esophagus (BE) is regarded as precancerous lesion of esophageal adenocarcinomas (EAC), some doubts have been raised regarding this association [7]. A substantial proportion of adenocarcinomas in the distal esophagus were not associated with Barrett mucosa. There are different potential explanations regarding pathogenesis and origin of these EAC without Barrett.

- First, AC without BE may have originated within a *Barrett mucosa*, which may have been *previously destroyed ('overgrown') *by the tumor [37,38]. It has been suggested, that neoadjuvant therapy may result in 'unmasking' of the previously 'overgrown' Barrett mucosa.

- Moreover, AC without BE may have originated in *very small spots of (ulta short segment) Barrett mucosa *or cases in which intestinal metaplasia was not stained with Cdx-2 [19].

- Finally AC without BE may have originated from another cell type, which might be the *putative cancer stem cell*.

A prognostic effect of LgR5 expression on protein level (IHC) was shown on univariate survival analysis. Patients with a high percentage of LgR5+ cells (>33%) exhibited a worse prognosis, in comparison to patients with lower LgR5+ staining. This was shown for the whole population of all patients with EAC under investigation, a result which is in line with previously published results [33]. We have furthermore shown, that a similar prognostic effect could be seen, when LgR5 expression was examined in a similar fashinon in adjacent Barrett's mucosa in EACs with BE. This result has not been decribed before and may be regarded due to the effect of 'field cancerization [39].

Taken together, LgR5-expression was found in a subset of proliferating LgR5+ intestinal cells, within BE, within BE-associated EAC as well as EAC without BE. Persistent activation of LgR5 in intestinal metaplasia and EACs may thus sustain multi-step carcinogenesis. Our findings seem to be very well in line with current understanding of carcinogenesis according to an integrated model of the CSC hypothesis and the clonal evolution theory [8]. Further investigations are required to substantiate these findings.

## Competing interests

The authors declare that they have no competing interests.

## Authors' contributions

VRBHA participated in the design of the study design, performed preliminary RT-PCR and immunohistochemistry studies and drafted the manuscript. All authors read and approved the final manuscript. SK participated in the design of the study, evaluated cancer samples, and helped to draft the manuscript. LM participated in the design of the study and performed RT-PCR studies. CR and LS participated in the design of the study, and performed immunohistochemistry studies. CO and GCT participated in the design of the study design and coordination and drafted the manuscript. GM conceived the study, carried out immunohistochemistry studies, performed the statistical analyzes and drafted the manuscript.
